# Optic nerve sheath diameter guided detection of sepsis-associated encephalopathy

**DOI:** 10.1186/s13054-020-03232-7

**Published:** 2020-08-25

**Authors:** Varun Suresh

**Affiliations:** Department of Anaesthesiology, Government Medical College, Thiruvananthapuram, Kerala 695011 India

I read with interest the research work done by Yang and colleagues [1] on measurement of ultrasonography-guided optic nerve sheath diameter (ONSD) in sepsis-associated encephalopathy (SAE) and its correlation with outcome. I appreciate the authors for successfully demonstrating raised ONSD in SAE patients; however, I have a few methodological concerns with the study.

The difference in ONSD across the three population groups compared in the study is sub-millimetric. Thus, an investigator not blinded to patient clinical condition can be biased to overestimate ONSD in SAE cases. This being a serious limitation authors could have sought for options of blinded ONSD measurements. It is also understood from the study results that elderly patients were included in the study population. A septated or trabeculated ONS being very common in elderly [2] such inclusion need not have essentially reflected the true ONSD.

I also wonder why a correlation between ONSD and serum albumin level was calculated. It is a very well-known fact that the blood-brain barrier is an osmotic interface [3] and oncotic pressure differences do not reflect on intracranial pressure. Do the authors essentially mean that hypoalbuminemia causes cerebral edema which indirectly reflects as high ONSD? It is inferred that this particular finding could be a statistical debate with zero clinical relevance.

Further, there is a considerable “blooming effect” artifact [4] in the ONSD images demonstrated by authors which could have affected the true ONSD measurements. Since majority of patients with SAE were on mechanical ventilation, a description of positive end-expiratory pressure and end-tidal carbon dioxide levels, which can directly affect ONSD [5], is distinctly wanted in the study results to exclude these confounders.

Unarguably Yang and colleagues’ effort on demonstrating ONSD threshold of ≥ 5.5 mm for detection of SAE is a novel contribution to knowledge, yet a narration on the concerns described here could have added more to the lucidity of author’s data.

## Data Availability

Not applicable
